# A Survey of Sodium Chloride Content in Italian Artisanal and Industrial Bread

**DOI:** 10.3390/foods7110181

**Published:** 2018-11-05

**Authors:** Marina Carcea, Valentina Narducci, Valeria Turfani, Altero Aguzzi

**Affiliations:** Research Centre for Food and Nutrition, Council for Agricultural Research and Economics (CREA-AN), Via Ardeatina 546, 00178 Roma, Italy; valentina.narducci@crea.gov.it (V.N.); valeria.turfani@crea.gov.it (V.T.); altero.aguzzi@crea.gov.it (A.A.)

**Keywords:** salt, sodium chloride, artisanal bread, industrial bread

## Abstract

A nationwide survey on salt content in both artisanal and industrial bread was undertaken in Italy to establish a baseline for salt reduction initiatives. Excess sodium intake in the diet is associated with high blood pressure and the risk of cardiovascular diseases. Bread has been identified as a major contributor to salt intake in the Italian diet. Most of the bread consumed in Italy comes from artisanal bakeries so 135 artisanal bread were sampled in 56 locations from Northern to Southern Italy together with 19 samples of industrial bread representative of the entire Italian production. Sodium chloride content was analysed according to the Volhardt’s method. A salt content between 0.7% and 2.3% g/100 g (as is basis) was found, with a mean value of 1.5% (Standard Deviation, 0.3). However, the majority of samples (58%) had a content below 1.5%, with 12% having a very low salt content (between 0.5% and 1.0%), whereas the remaining 42% had a salt content higher than the mean value with a very high salt content (>2.0%) recorded for 3% of samples. As regards the industrial bread, an average content of 1.6% was found (SD, 0.3). In this group, most of the samples (56%) had a very high content between 2.0% and 2.5%, whereas 5% only had a content between 1.1% and 1.5%. Statistics on salt content are also reported for the different categories of bread.

## 1. Introduction

One third of global deaths are due to cardiovascular diseases, including heart attacks, strokes and related diseases (World Health Organization, 2007). High blood pressure is the major risk factor and, according to a substantial body of epidemiological and interventional studies, an excess of sodium in the diet is the primary cause of hypertension [1,2,3,4,5]. Salt intake is thus being increasingly monitored and evaluated worldwide. The human physiological need of sodium is rated around 130–230 mg/day by the World Health Organization (WHO), but in many industrialized countries sodium intake is actually 3600–4800 mg/day [6]. This indicates that the mean salt intake of populations is well in excess of dietary needs and far from the WHO recommendation to have a salt intake <5 g/day [6], that is, 2000 mg/day of sodium.

In the last decades, a wide range of initiatives aimed at salt reduction (DASH: Dietary Approaches to Stop Hypertension, WASH: World Action on Salt and Health, National Salt Reduction Weeks, CASH: Consensus Action on Salt and Health) have been started at the international level to sensitize people about salt consumption and salt content in some food categories, to educate the population about the dangers of salt in excess, and to translate scientific evidence into public health policies and plans for reformulation of processed foods [1,3,5,7,8,9,10,11]. In fact, processed foods are the main source of salt in the diet, with cereal products contributing the most of the overall intake [6,10,12,13], especially in those countries where bread is consumed daily at every meal. A recent survey highlighted an average yearly consumption per capita of 64 kg in Europe with Italy ranking third after Germany and France (57 kg) [14].

When in 2008 the European Commission (EC) launched the EU Framework for National Salt Initiatives, an interdisciplinary Working Group for reduction of salt intake (GIRCSI) was established in Italy at the Ministry of Health [3] with the main objective to device strategies to reduce salt consumption in the population. Bread was identified as one of the first processed foods to address and the first steps to be taken were to measure and monitor the sodium content of bread to promote reformulation of foods containing less salt. Other European countries have launched initiatives to reduce salt content in bread and recently news has appeared on the Internet that Portugal will set mandatory maximum salt levels in bread by 2019 [15].

This paper represents the first comprehensive survey on the salt content in bread consumed by the Italian population, and the data reported here represent the baseline for the reformulation of salt reduced bread. Most of the bread consumed in Italy is produced by artisans in artisanal bakeries according to different recipes and procedures and only a small proportion of the market (around 10%) is covered by the industrial production: consequently, a great variability in salt content was expected. Several breads in Italy are also protected by European authenticity labels such as Protected Designation of Origin (PDO) and Protected Geographical Indication (PGI) labels. Both artisanal and industrial bread was considered in the present study. Moreover, a comparison between methods to determine Na content in flour and bread was made on selected bread samples to assess the reliability of the quick method which was used for sodium chloride determination in bread.

## 2. Materials and Methods

### 2.1. Samples and Sampling Method

Artisanal bread was purchased at selected bakeries in Northern, Central and Southern Italy particularly in places with a specific identity in terms of bread production. In each bakery, the most consumed types of bread were sampled. For the industrial sector, samples of all the Italian production available on the market were purchased at supermarkets and included sliced pan bread (12 samples) and “traditional-like” bread (6 samples). In total, 154 bread samples (kinds of bread) were collected, between winter 2009 and spring 2010. For each type of sample, a spreadsheet was filled with data concerning origin, ingredients, weight and baking method.

**Figure 1:**
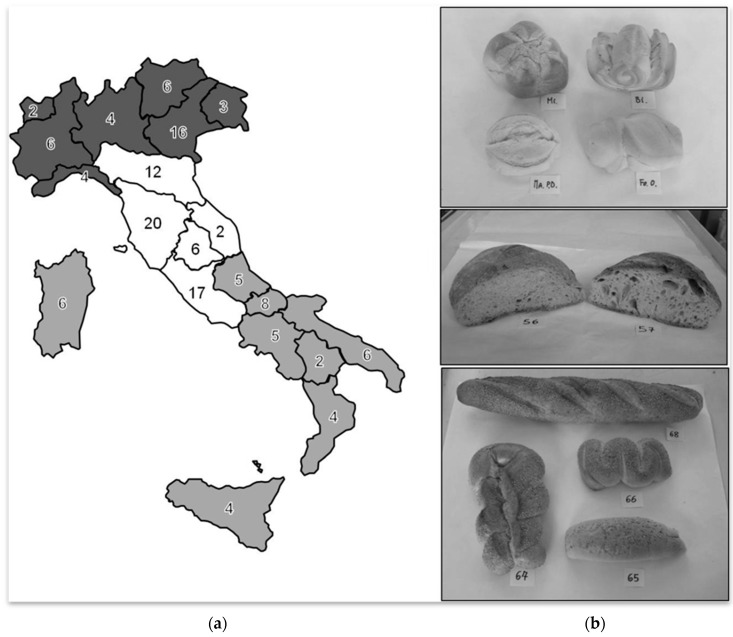
Bread samples collected in the different regions of Northern, Central and Southern Italy. (**a**) Number of samples from each Italian region. Northern regions are coloured in dark grey, Central regions in white and Southern regions in light grey (division according to the Italian Central Institute of Statistics). (**b**) Bread samples of different size, shape and ingredients.

In detail, 19 samples of industrial bread were collected together with 135 samples of artisanal bread from 56 locations (Figure 1). Seven out of 154 samples (1 sample of industrial bread and 6 samples of artisanal bread) were declared, at purchase, without salt and subsequent analysis performed by us, confirmed this feature.

Samples of baking wheat flour (*Triticum aestivum* L. flour, which is the kind of flour mostly used in bread baking in Italy) of two different extraction rates according to the Italian law (0 and 00, ash content maximum 0.65% and 0.55% on dry matter, respectively) were purchased at a local supermarket and analysed for their sodium content.

### 2.2. Analytical Methods

Soon after purchase, representative portions of each type of bread were cut in small pieces, well homogenised and used for the following analyses. A portion of the sample was used to determine moisture according to ICC Standard No. 110/1 [16], whereas another portion was prepared according to AACC method 62-05 “Preparation of sample: bread” [17] by drying it at 35 °C overnight and grinding it by a MLI 204 laboratory mill (Bühler, Uzwil, Switzerland). The residual moisture in the sample was also determined according to the previous ICC Standard. The determination of chloride ion in bread samples was carried out by titration according to the AACC method 40-33 “Chloride in yeast foods—quantitative method (Volhardt’s method)” [17]. Sodium chloride content was finally calculated based on the content of chloride ions in sample. Duplicate analysis was carried out for each sample. Duplicates differing by more than 0.20 were rejected and analysis repeated. Salt content in bread was expressed as percentage, as is basis.

A selection of bread and wheat flour samples were also analysed by Inductively Coupled Plasma Spectroscopy (ICP) on a Perkin-Elmer Plasma Optima 3200XL (Perkin-Elmer, Waltham, MA, USA) in order to determine sodium content in the raw material, and confirm that the results obtained by the AACC method were in good match with those obtained by ICP. Samples were first mineralized in nitric acid (6 mL HNO_3_ + 1 mL H_2_O_2_) in a microwave oven (Milestone 1200 Mega, FKV srl, Torre Boldone, Italy). Standard CRM 189 (whole meal flour) from the Community Bureau of Reference (BCR, Brussels, Belgium) was used as a Reference Material.

### 2.3. Statistics

The seven samples of bread without salt were excluded from statistical elaboration. Statistical determination of mean, standard deviation and percentage distribution were performed using Microsoft Office Excel 2007. For easiness of results understanding and interpretation, it was decided to establish 4 classes of salt content (as is basis): (i) 0.5–1.0% (low salt content); (ii) 1.1–1.5% (medium salt content); (iii) 1.6–2.0% (high salt content); and (iv) 2.1–2.5% (very high salt content).

The percentage distribution in the above-mentioned salt content classes was calculated for 14 groups that represented all the different commercial categories that could be found in our sample population: all samples together, industrial vs. artisanal samples, 4 categories according to weight, 5 categories according to ingredients, and 2 categories according to leavening method.

## 3. Results

This section presents the results of analyses of sodium content in soft wheat white flour widely used for bread baking in Italy, and eight samples, selected for their different characteristics and presumably different salt content, are reported in Table 1. The same table briefly describes each sample compositional or processing characteristics. One column reports data obtained by calculating the sodium content in samples analysed by the standard AACC method 40-33 (Volhardt’s method) [17], whereas the other column refers to the sodium content in samples determined by means of ICP.

The purpose of this study was to assess the contribution of the raw material flour to the salt content in bread, to verify whether the bread declared to be without any salt actually had a negligible sodium content, and whether the data obtained by the Volhardt’s method could be compared with those obtained by a more sensitive but more complex and expensive method.

Data reported in Table 1 show that sodium was not detected in both types of commercial soft wheat white flours, even in the 0 type which is less refined than 00. Based on this result obtained with a very sensitive instrument, it was decided not to analyse these two samples by the Volhardt’s method.

No sodium was detected following both analytical procedures in the three different bread samples declared by the bakers to be without salt addition. Sodium was detected by means of both methods in the five remaining samples and values ranged 03–06 for the Volhardt’s method and 0.1970–0.4902 g/100 g (as is sample) for the ICP method. In both cases, the highest value was obtained for durum wheat bread.

**Figure 2:**
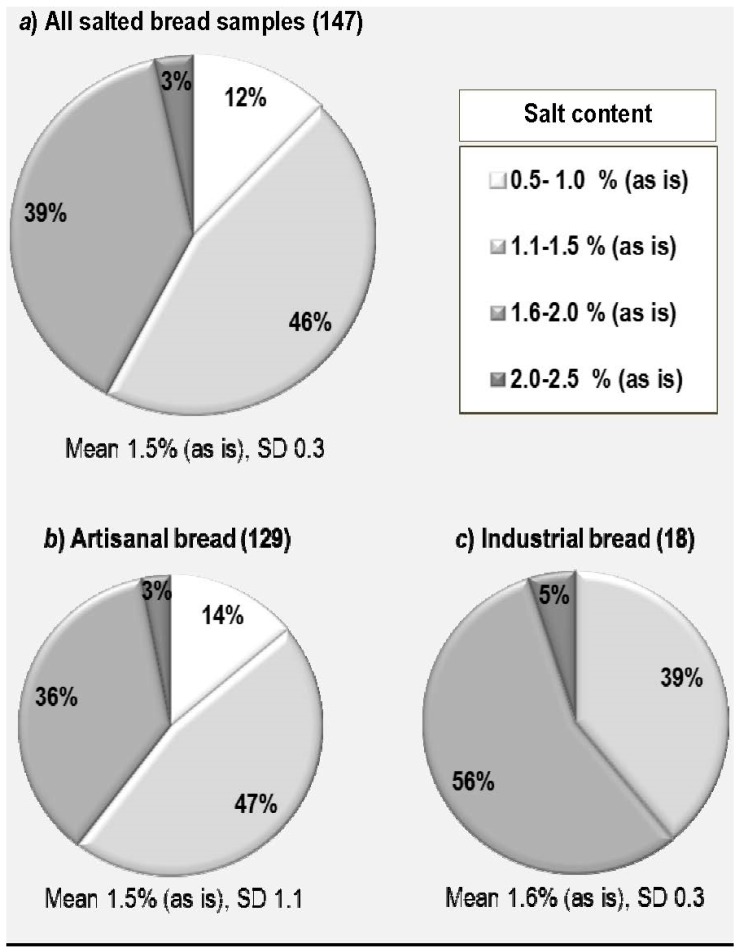
Percent distribution of bread samples according to salt content classes.

**Figure 3:**
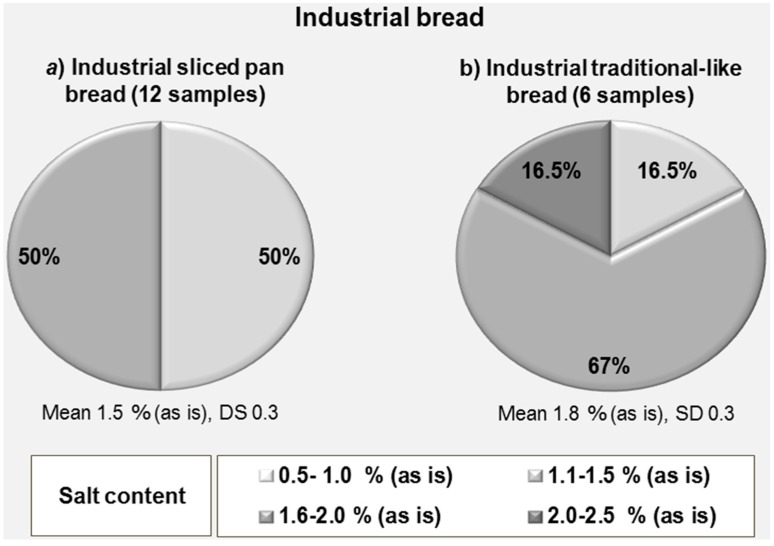
Percent distribution of industrial bread samples according to salt content classes.

**Figure 4:**
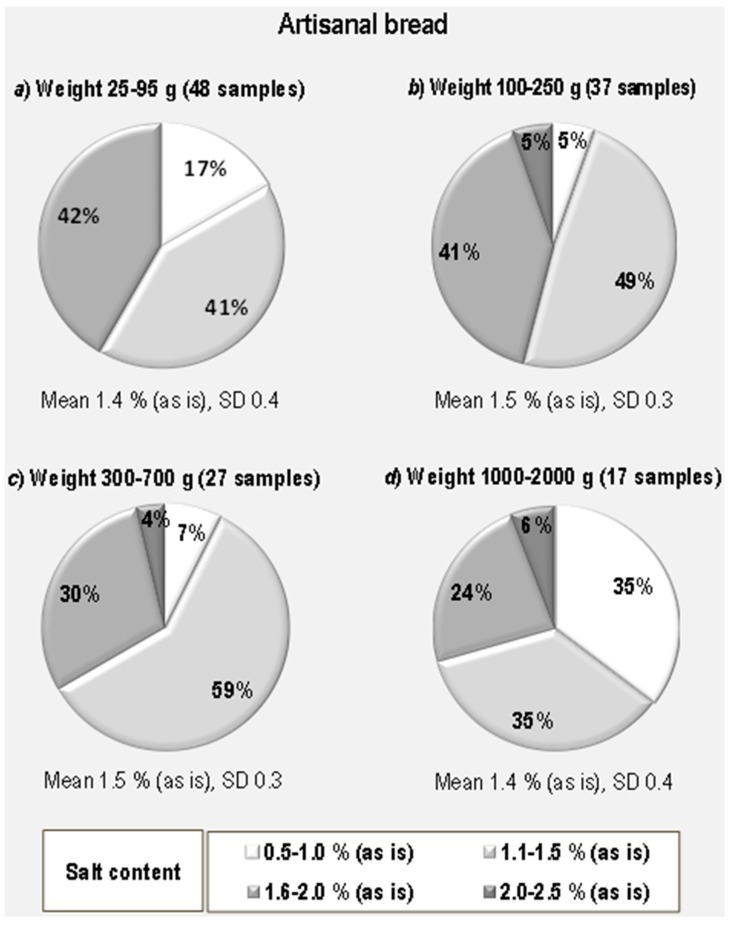
Percent distribution of artisanal bread samples of different weight according to salt content classes.

**Figure 5:**
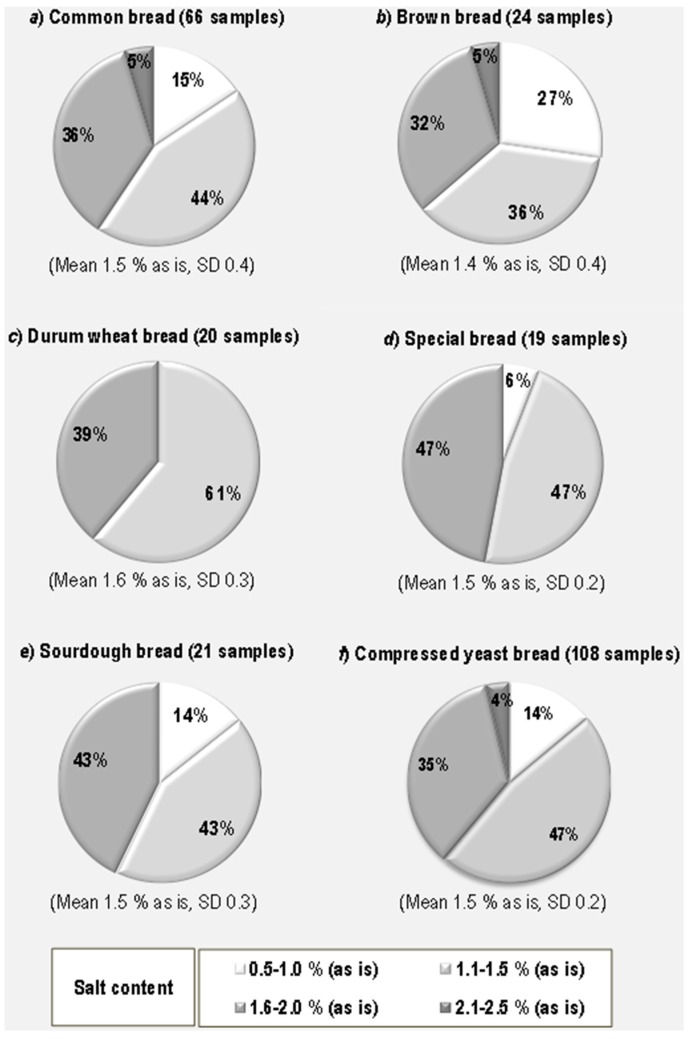
Per cent distribution of artisanal bread samples, differing in dough formulation and leavening method, according to salt content classes.

The statistical elaboration of salt content data referring to the 147 samples of salty bread is reported in Figure 2, Figure 3, Figure 4 and Figure 5. In our survey, a salt content in bread ranging between 0.7% and 2.3% (as is basis) was found, with a mean value of 1.5% and a standard deviation (SD) of 0.3 (Figure 3). If we look at the distribution of salt content in the different classes as specified in the Materials and Methods Section, we can see that the majority of bread samples (58%) had a salt content below the reported mean value (>1.5%) (Figure 2a) with 12% having a very low salt content falling within the range 0.5–1.0%, whereas the remaining 42% had a salt content higher than the mean value with a very high salt content (>2.0%) recorded for 3% of samples.

If we have a separate look at the artisanal and the industrial production (Figure 2b,c), we can say that, although the average salt content in bread is very similar (1.5 and 1.6 g/100 g as is basis with standard deviations of 1.1 and 0.3, respectively), the distribution of our samples in the different salt content classes is different. In the artisanal bread, the majority of bread samples (61%) had a salt content below the reported mean value (>1.5%) (Figure 2a) with 14% having a very low salt content falling within the range 0.5–1.0%, whereas the remaining 39% had a salt content higher than the mean value with a very high salt content (>2.0%) recorded for only 3% of samples. In the industrial production, only three classes were represented, the very low salt content class (<1.0 g/100 g as is) having disappeared. Most of the samples (56%) had a very high salt content between 2.0 and 2.5 g/100 g (as is) whereas only 5% had a salt content between 1.1 and 1.5 g/100 g (as is).

A further differentiation can be made within the industrial bread by considering separately the sliced pan bread, which represents the most consumed category, and the so-called “traditional-like” bread which resembles more in its shape the artisanal bread (Figure 3). In the pan bread, a mean value of 1.5 g/100 g (as is) was obtained (SD 0.3) and two salt content classes (1.1–2.0%) were found, each having a 50% share, whereas in the traditional-like bread an average value of 1.8% g/100 g (as is) was found (SD 0.3) which derived from the contribution of three salt content classes (1.1–2.5%) with the very high salt content class having a share of 16.5%.

Given the great variety of artisanal bread, we thought it would be interesting to compare the salt content in different types of bread to determine whether there was any relationship between specific bread characteristics and salt content: weight, ingredients and leavening method were identified as interesting quality traits. The 129 artisanal bread loaves were, therefore, grouped into three different categories according to their weight, ingredients and leavening method. Within the “weight” category, four classes were identified based also on the bread shape: (i) 25–95 g (48 samples); (ii) 100–250 g (37 samples); (iii) 300–700 g (27 samples); and (iv) 1000–2000 g (17 samples), with rolls, typical of the bread production in Northern Italian regions, and big loaves typical of Central and Southern regions. Four classes were also established in the “ingredients” category as follows: common white bread, whose dough is typically formulated with just soft wheat flour, water and salt (66 samples); brown bread, with different amounts of soft wheat whole-meal flour in addition to the common white bread ingredients (24 samples); durum wheat bread (20 samples), typical of Southern Italy but also appreciated and consumed all over Italy made with remilled durum wheat semolina, water and salt; and “special” bread, that is, soft wheat white bread with other ingredients such as oil, milk, and potatoes (19 samples). As regards the leavening method, two classes were established: sourdough and compressed yeast. 

Figure 4 reports the pie charts of the percentage distribution in the four salt content classes according to the weight of the bread. The most represented weight class was small breads, i.e., rolls (48 samples), and the least represented was big loaves weighing up to 2 kg. This distribution actually reflects the pattern of consumption of the Italian population. The categories up to 250 g were the most represented. Although the average salt content and SD is very similar or identical in the four groups and goes from 1.4 to 1.5 g/100 g (as is), (SD, 03 and 0.5, respectively), the percentage distribution of the four salt content classes was different and peculiar within each group with the highest salt content class not being represented for example in the smallest bread group and the biggest loaves having the highest percentage of samples (6%) having a salt content between 2.0% and 2.5% (as is).

For dough formulation (Figure 5), we obtained a mean value of 1.4 g/100 g (as is) (SD, 0.4), for brown bread, 1.5 g/100 g (as is) (SD, 0.4 and 0.2, respectively, for common bread and special bread), and for durum wheat bread, 1.6 g/100 g (as is) (SD, 0.3). In the durum wheat group, only two salt classes were found, namely 1.1–1.5% and 1.6–2.0%, with the first being more represented (61%) than the latter (39%).

The two leavening methods had very different sizes, with sourdough samples being only 21 while compressed yeast bread samples being 108. These numbers actually reflect the presence of these categories on the market with sourdough bread being less frequently found. However, the two groups had the same average salt content, 1.5 g/100 g (as is) (with SD = 0.3 for sourdough bread, and SD = 0.2 for compressed yeast bread). In the sourdough bread group, there were no samples with a very high salt content (≥2.1%).

## 4. Discussion

Recently, several similar surveys have been conducted in countries where bread is a staple food and has therefore been identified as a major contributor to the daily intake of salt and sodium in the population [18,19,20,21].

In our study, the analysis of sodium content in a selection of commercial refined wheat flour and bread samples by ICP analysis showed that salt content in white bread, which is the most consumed type of bread in Italy, is not due to a natural occurrence of sodium in the flour, but to the salt added in the recipe. The higher sensitivity of the ICP analysis than the Volhardt’s method enabled to confirm, in fact, that sodium naturally occurring in the white flour is negligible (Table 1) and, moreover, it showed that salt content in some of the sampled bread samples, declared at purchase to be “without salt”, was, in fact, below 0.1%.

Even if there is no perfect correspondence between the results obtained by the two methods (Table 1), it is nevertheless interesting to notice that the ranking of the samples as regards their sodium content was the same. These results confirmed the practical value of the Volhardt’s method for the determination of sodium chloride in bread and for the purpose of our study.

Although the average salt content found in all our bread samples (1.5% g/100 g, as is basis) is similar to that reported in the literature for other European countries [22], the range of values found was very wide with the highest values around 2.3%. This means that there is room for improvement and that salt reduction initiatives and campaigns are advisable also in Italy.

The statistical elaboration of data also showed an interesting variation of salt content in bread at geographical level. It emerged that the mean salt content in bread produced and consumed in Central Italy is slightly lower than in the north and south of the country. In fact, the mean salt content was 1.3% in the 52 bread samples from Central Italy with a SD of 0.4, whereas it was 1.6% with a SD of 0.2 in the 38 bread samples from Northern Italy, and 1.5% with a SD of 0.3 in the 39 bread samples from Southern Italy. In detail, it emerged that in Northern Italy there is no share of bread with a salt content below 1.0%, whereas 21% of analysed samples purchased in Central Italy and 15% of bread types sampled in Southern Italy were in this range. These figures confirm the existence of a well-established tradition in some regions of central Italy, e.g., Umbria, Marche and Tuscany, of producing bread loaves with a very low or null salt content. This evidence also hints at the fact that the main problem in salt reduction might be consumers’ acceptance and salt content in bread might be reduced at the artisanal level without encountering too many technological problems.

Considering separately the artisanal production from the industrial production, even though in Italy the latter represents one fourth of the former, it is interesting to notice that the average salt content is higher in industrial bread (1.6% g/100 g, as is basis, with a SD of 0.3) than in the artisanal bread (1.5% g/100 g, as is, and a wider SD 1.1). and no samples were found falling within the class containing a small amount of salt (0.5–1.0%). Most industrial samples (56%) fall in the high salt content class (1.6–2.0%), whereas artisanal bread’s most represented category (47%) is that of 1.1–1.5% salt content (medium salt content). The industrial production can easily be subdivided into two categories, namely pan bread (which is always sliced) and traditional-like bread which is more similar in shape and appearance to artisanal bread. They represent the two most common types of industrial bread that are produced by a few manufacturers in a homogeneous and standardized way, and distributed all over the national territory. It is interesting to notice that the pan bread had a more homogeneous salt content, ranging from 1.1% to 2.0% with an average of 1.5%, as is, and a SD of 0.3, whereas the traditional-like bread had 16.5% of samples having a salt content between 2.1% and 2.5% and a higher average content of 1.8% and the same value (0.3) of standard deviation.

The average content in Italian industrial bread is higher than that reported in other European countries such as UK, where in 2011 a National survey, promoted by the Consensus Action on Salt and Health (CASH), reported for industrial pre-packaged bread a salt content ranging between 0.58% and 0.83% [7].

In addition, in the industrial Italian production, it is advisable to reduce the salt content and, considering that most of the production is in the hands of few manufacturers, it should not be too difficult to reach this target. Moreover, being industrial bread generally supplied to canteens, hospitals and caterings, there are high chances that salt reduction initiatives can reach a broad number of consumers in a very short time even if the artisanal market share represents the biggest challenge for any future salt reduction initiative.

The analysis of salt content in bread according to its weight showed two significant pieces of evidence. In big loaves weighing 1000–2000 g (Figure 4d), there is a more consistent percentage of samples (35%) with a very low salt content (0.5–1.0%, as is basis). On the other hand, rolls weighing 25–95 g (Figure 4a) proved to be the only weight class with a salt content always below 2.0% and never reaching the very high content. Comparing the results obtained for the four classes under consideration with the mean salt content obtained for artisanal bread (1.5%, as is basis, with SD of 1.1), it emerged that a very good share of samples for each class has values below this mean: 57% of rolls (class 25–95 g), 54% of small loaves (class 100–250 g), 66% of medium loaves (class 300–700 g) and 70% of big loaves (class 1000–2000 g).

Considering dough formulation, i.e., the different raw materials used in bread making (Figure 5), it emerged that durum wheat bread had a more homogeneous salt content than common, brown or special bread: all samples belonged to only two salt classes, namely 1.1–1.5% and 1.6–2.0%. The main share (61%) is due to the lower salt content class. Considering the mean value of salt content in artisanal bread as a reference point for discussion, it was observed that 59% of common bread (Figure 5a), 63% of brown bread (Figure 5b), 61% of durum wheat bread (Figure 5c) and 53% of special bread (Figure 5d) samples have a salt content lower than this mean.

Bread samples with a salt content exceeding 2% belonged only to the class “common bread” and “brown bread”, but at the same time brown bread is the category with the highest percentage (27%) of samples with a very low salt content (0.5–1.0%, as is basis) followed by common bread (15% of samples). The main difference between the sourdough and compressed yeast bread categories can be seen in the presence of 4% samples with a very high salt content (2.1–2.5%). The average content is the same for both categories, i.e., 1.5%, as is, but the SD is higher (0.3 versus 0.2) for sourdough bread. By focusing on the results obtained for the sourdough bread and brown bread categories, which had a significant percentage of the very low salt content, it could be speculated that the use of the sourdough and the formulation with wholemeal flours, can add to bread a natural flavour that prevents an excessive addition of salt to the dough.

## 5. Conclusions

The present study represents the first extensive survey on the actual salt content in Italian bread and provides the baseline for national salt reduction initiatives, as recommended by the European Commission (EC) to each country within the EU Salt Reduction Framework [8].

As regards artisanal bread, which is the type of bread mostly consumed by the Italian population, the survey highlighted a great variability of values obtained for salt content (from 0.7% to 2.3%, as is basis) that enabled both the identification of a market share offering bread with a high-salt content (2.0–2.5%) that should be immediately addressed by salt reduction policies and education campaigns, as well as the existence of a substantial share of bread with a low salt content that is in line with the EC and WHO recommendations. A good share of the Italian bakery market is represented by the long-established tradition of bread produced with a low salt content (0.5–1.0%) and widely consumed in some regions of Central Italy, e.g., Marche, Toscana and Umbria. This evidence indicates that technological strategies for low-salt bread manufacturing and campaigns for consumer education to gradual salt reduction in bread are possible with high chances of success.

As regards industrial bread, there is less variation in salt content compared to artisanal bread but it is on the high content side. However, future initiatives for salt reduction are more likely to be successful and reach in shorter times a major share of consumers because industrial bread production is controlled by a few manufacturers that distribute their standardized products all over Italy.

## Figures and Tables

**Table 1:** Sodium content in flour and bread samples as measured by two methods of different sensitivity.

Sample	Sodium Content Volhardt * (g/100 g)	Sodium Content ICP * (g/100 g)
Commercial white flour (Italian type 00)	not analysed	not detected
Commercial white flour (Italian type 0)	not analysed	not detected
Sample 1 (bread without salt, 500 g)	not detected	not detected
Sample 4 (bread without salt, 500 g)	not detected	not detected
Sample 14 (bread without salt, 500 g)	not detected	not detected
Sample 32 (common bread, 95 g)	0.3	0.2249
Sample 38 (wholemeal sourdough bread, 1.5 kg)	0.4	0.3283
Sample 25 (durum wheat bread, 170 g)	0.6	0.4902
Sample 57 (sourdough bread, 2 kg)	0.3	0.1970
Sample 67 (special bread, 200 g)	0.6	0.4552

* Average of two determinations on as is sample.
